# Insulin resistance estimated by estimated glucose disposal rate predicts outcomes in acute ischemic stroke patients

**DOI:** 10.1186/s12933-023-01925-1

**Published:** 2023-08-26

**Authors:** Zhengzhao Lu, Yunyun Xiong, Xueyan Feng, Kaixuan Yang, Hongqiu Gu, Xingquan Zhao, Xia Meng, Yongjun Wang

**Affiliations:** 1 Department of Neurology, Beijing Tiantan Hospital, Capital Medical University, Beijing, China; 2 China National Clinical Research Center for Neurological Diseases, Beijing, China; 3 Chinese Institute for Brain Research, Beijing, China; 4 National Center for Healthcare Quality Management in Neurological Diseases, Beijing, China; 5 Research Unit of Artificial Intelligence in Cerebrovascular Disease, Chinese Academy of Medical Sciences, 2019RU018, Beijing, China

**Keywords:** Insulin resistance, Ischemic stroke, Outcome, Secondary prevention, Estimated glucose disposal rate

## Abstract

**Background:**

Estimated glucose disposal rate (eGDR), a simple and noninvasive measure of insulin resistance, has been proven to be an independent risk factor for first-time stroke and all-cause mortality. In this study, we aimed to investigate the associations between eGDR and the stroke outcome in patients with first-time acute ischemic stroke (AIS).

**Methods:**

We included first-time AIS patients with available data on eGDR in the China National Stroke Registry III (CNSR-III), and divided the subjects into lower eGDR group (eGDR ≤ 6 mg/kg/min) and higher eGDR group (eGDR > 6 mg/kg/min). The primary outcome was excellent functional outcome (modified Rankin Scale score 0–1) at 3 months. Secondary outcomes included stroke recurrence and favorable functional outcome (modified Rankin Scale score 0–2) at 3 months, and functional outcome and combined vascular event at one year. Univariate and multivariate analyses were performed to evaluate the association between eGDR and outcomes.

**Results:**

A total of 6,271 patients with AIS were included in this study. The median values of eGDR in lower and higher eGDR group were 5.0 mg/kg/min (interquartile range, 4.2–5.6) and 7.6 mg/kg/min (interquartile range, 6.8–9.6), respectively. Patients with higher eGDR were significantly associated with higher incidence of excellent functional outcome (adjusted odds ratio, 1.24; 95% confidence interval, 1.06–1.45; P < 0.01) at 3 months and favorable (adjusted odds ratio, 1.55; 95% confidence interval, 1.24–1.93; P < 0.01) and excellent (adjusted odds ratio, 1.28; 95% confidence interval, 1.08–1.51; P < 0.01) functional outcome at one year. However, there was no significant difference in stroke recurrence between these two groups at 3 months (adjusted odds ratio, 0.81; 95% confidence interval, 0.61–1.06; P = 0.12) and one year (adjusted odds ratio, 0.91; 95% confidence interval, 0.73–1.14; P = 0.41).

**Conclusion:**

eGDR is a predictor of functional outcome in patients with AIS, independent of traditional cardiovascular predictors.

**Supplementary Information:**

The online version contains supplementary material available at 10.1186/s12933-023-01925-1.

## Introduction

Stroke is one of the major causes of death and disability worldwide [1], and imposes a heavy economic burden globally [2]. Ischemic stroke accounts for approximately 80% of all strokes and is associated with many risk factors such as hyperlipidemia [3], diabetes [4], hypertension [5] and insulin resistance [6].

Insulin resistance occurs commonly in type 2 diabetes (T2D) and about 50% of acute ischemic stroke (AIS) patients without diabetes [7]. It is considered as a new independent predictor of first-time AIS [8], and related with higher rates of disability in AIS patients [9]. The Insulin Resistance Intervention after Stroke (IRIS) trial showed that pioglitazone which improves insulin sensitivity could decrease the risk of AIS or myocardial infarction in nondiabetic patients with a recent transient ischemic attack (TIA) or AIS [10].

In addition, some studies showed that insulin resistance was also related to some independent risk factors of poor stroke outcomes such as hypertension [11], hypertriglyceridemia and lower high-density lipoprotein (HDL) [12]. The interaction between insulin resistance and these factors makes it difficult to evaluate the independent influence of insulin resistance on stroke outcomes, and might require a large sample size to reduce confounding. According to a mathematical analysis, intervention targeting insulin resistance could prevent approximately 42% of cardiovascular disease cases. Additional therapy, such as use of hypotensive and antidiabetic drugs, may further reduce the risk [13].

Currently, the gold standard test diagnosing insulin resistance is hyperinsulinemic-euglycemic clamp. However, it is costly, invasive and unavailable in the real-world practice [14]. Therefore, some noninvasive methods were developed to measure insulin resistance such as homeostasis model assessment-insulin resistance (HOMA-IR) [15], triglyceride glucose index (TyG index) [16] and estimated glucose disposal rate (eGDR) [17].

eGDR was originally developed as a validated score to measure insulin resistance in type 1 diabetes (T1D) based on waist circumference, hypertension and glycated hemoglobin A (HbA1c) [8]. Compared with hyperinsulinemic-euglycemic clamp, this method has a higher accuracy [18] and is suitable for clinical practice and a large cohort study. Recently, some studies found that higher eGDR was associated with a decreased risk of AIS, all-cause mortality and cardiovascular disease in patients with T1D [19] and T2D [8]. However, the relationship between eGDR and stroke outcomes in AIS patients is unknown. In this study, we aimed to investigate the associations between insulin resistance evaluated by eGDR formula and AIS recurrence, functional outcome and combined vascular event in patients with first-time AIS.

## Methods

This study was approved by the Central Institutional Review Board in Beijing Tiantan Hospital (IRB approval number: KY2015-001-01). Written informed consents were obtained from all participants or legally authorized representatives for vulnerable participants.

### Study population

This was a retrospective cohort sub-study of the China National Stroke Registry III (CNSR-III). The protocol of this registry has been reported previously [20]. Briefly, CNSR-III was a prospective, nationwide registry enrolling AIS or TIA patients in China from August 2015 to March 2018.

**Fig. 1:**
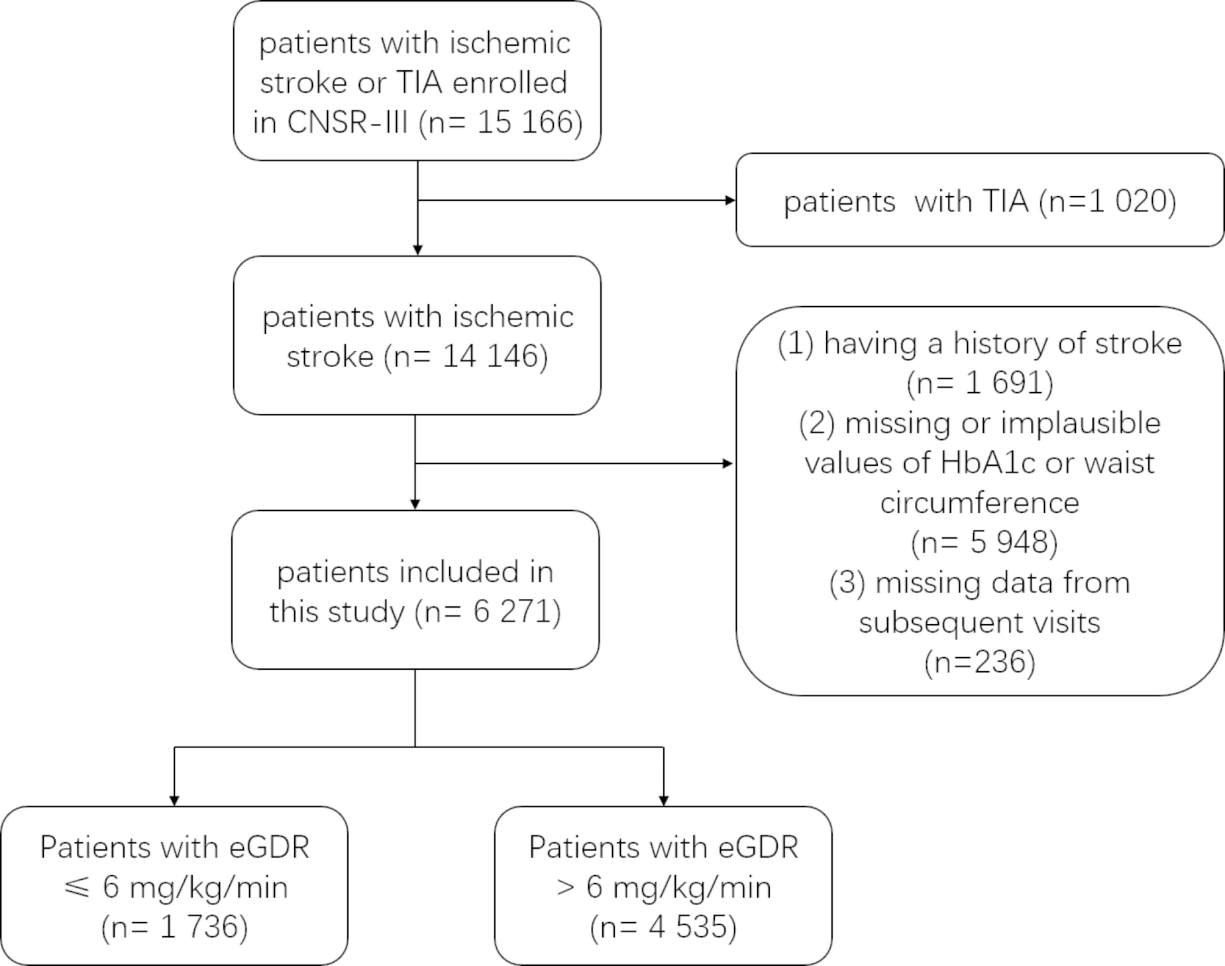
Flowchart of Patient Recruitment TIA, transient ischemic attack; CNSR-III, The Third China National Stroke Registry; HbA1c, hypertension and glycated hemoglobin A; eGDR, estimated glucose disposal rate

Patients above 18 years old with AIS within 7 days from onset of symptoms were included in this study. Exclusion criteria were: [1] history of stroke [2], missing or implausible data of HbA1c or waist circumference at baseline [3], missing data from subsequent follow up visits. A flow chart of patients can be seen at Fig. 1.

### Data collection

Demographics, medical history, laboratory tests and other clinical characteristics of all participants were collected. The 3-month and one-year follow-up are conducted through face-to-face interviews. Information including health status, use of medications, occurrence of stroke or other cardiovascular events was queried at each follow-up.

Renal insufficiency was defined as glomerular filtration rate (GFR) < 60 mL/min/1.73 m^2^. The National Institutes of Health Stroke Scale (NIHSS) score was evaluated by trained researchers at admission. Body Mass Index (BMI) was calculated by using the following formula: weight (kg) / [height (m)]^2^. eGDR (mg/kg/ min) was used as a measure of insulin resistance and calculated at baseline by using the following formula [18]:

$$\begin{array}{l}{\rm{eGDR}}\,{\rm{ = }}\,{\rm{21}}{\rm{.158}}\, - \,\left( {{\rm{0}}{\rm{.09*WC}}} \right) - \left( {{\rm{3}}{\rm{.407*HT}}} \right)\\- \left( {{\rm{0}}{\rm{.551*HbA1c}}} \right)\,[{\rm{WC = }}\,{\rm{waist}}\,{\rm{circumference}}\left( {{\rm{cm}}} \right){\rm{,}}\,\\{\rm{HT}}\,{\rm{ = }}\,{\rm{hypertension}}\,\left( {{\rm{yes}}\,{\rm{ = }}\,{\rm{1/no}}\,{\rm{ = }}\,{\rm{0}}} \right){\rm{,}}\,\\{\rm{and}}\,{\rm{HbA1c}}\,{\rm{ = }}\,{\rm{HbA1c}}\,\left( {\rm{\% }} \right)]\end{array}$$

Waist circumference was measured by using the natural waist location. Hypertension was defined as blood pressure higher than 140/90 mmHg on two occasions or using of antihypertensive medication.

### Study outcomes

The primary outcome of this study was excellent functional outcome at 3 months, defined as a modified Rankin Scale (mRS) of 0–1. Secondary outcomes included favorable functional outcome at 3 months, defined as a mRS of 0–2, excellent functional outcome at one year, ischemic stroke recurrence at 3 months and one year, and combined vascular events at one year. Combined vascular events were defined as myocardial infarction, ischemic stroke, hemorrhagic stroke, subarachnoid hemorrhage or cardiovascular death.

### Statistical analysis

Continuous variables at baseline were described as mean ± standard deviation (SD), and categorical variables were described as frequency (percentage). We divided AIS patients into lower eGDR group (eGDR ≤ 6 mg/kg/min) and higher eGDR group (eGDR > 6 mg/kg/min). We selected a cut-off value of 6 mg/kg/min based on the distribution of our data, which closely approximated the 25% quartile of 5.8 mg/kg/min. Differences in demographic and clinical variables were compared using Student’s t-test and Chi-square tests according to types and normality of the data. Subsequently, three logistic multivariate regression analysis models were conducted to investigate the association between eGDR and functional outcome, combined vascular event and ischemic stroke recurrence. Model 1 was unadjusted. Model 2 was adjusted for demographic parameters (age and sex). Model 3 was adjusted for demographic parameters and imbalanced baseline variables including T1D, T2D, lipid metabolism disorder, coronary heart disease, atrial fibrillation, infection within 2 weeks before admission, sleep apnea, drinking, BMI, HDL, triglycerides, thrombolysis, hypoglycemic therapy, antiplatelet therapy, and anticoagulant therapy. We reported odds ratios (ORs) and 95% confidence intervals (CI). Considering that HbA1c, WC, hypertension, and antihypertensive therapy are part of the eGDR formula, these variables were excluded in the logistic multivariate regression model. Restricted cubic spline in a logistic regression model was conducted to investigate the linear relation between eGDR and excellent outcome at 3 months. Subgroups analyses were conducted for subgroups of age, sex, diabetes, thrombolysis and stroke subtypes.

All P values were two-sided. P < 0.05 was considered statistically significant. All analyses were performed with SAS V.9.4 software (SAS Institute, Inc, Cary, NC).

## Results

### Study population and baseline clinical characteristics

Among 14,146 patients with AIS, 1,691 patients were excluded due to their history of stroke, 5,948 patients were excluded due to missing or implausible values of HbA1c or waist circumference, 236 patients were excluded due to missing data from subsequent visits. Finally, A total of 6,271 patients were included in this study (Fig. 1). At baseline, 68.1% of the cohort were male and the average age is 61.5 ± 11.4 years. 32% of the patients were diagnosed with T1D or T2D, and 36% of the patients were diagnosed with abnormal glycemia at discharge. Abnormal glycemia is defined as abnormal glucose tolerance, impaired fasting glucose, T1D and T2D. The patients were divided into lower eGDR group (n = 1,736, 27.7%) (eGDR ≤ 6 mg/kg/min) and higher eGDR group (n = 4,535, 72.3%) (eGDR > 6 mg/kg/min). The median values of eGDR in lower and higher eGDR group were 5.0 mg/kg/min (interquartile range, 4.2–5.6) and 7.6 mg/kg/min (interquartile range, 6.8–9.6) respectively. Baseline demographic, clinical characteristics and differences between groups were displayed in Table 1. Compared with higher eGDR group, lower eGDR group patients were more likely to suffer from diabetes, hypertension, lipid metabolism disorder, and coronary heart disease. More patients in higher eGDR group received thrombolysis therapy at baseline and more patients in lower eGDR group received antiplatelet therapy at 3-month follow-up visit.

**Table 1:** Demographics and clinical characteristics of patients with first-time acute ischemic stroke

Variables	Total	eGDR ≤ 6 mg/kg/min	eGDR > 6 mg/kg/min	P Value
	(N = 6271 [100%])	(N = 1736 [27.7%])	(N = 4535 [72.3%])	
**Age, Mean ± SD, y**	61.5 ± 11.4	61.0 ± 10.9	61.8 ± 11.5	0.02
**Male, n (%)**	4271 (68.1)	1210 (69.7)	3061 (67.5)	0.10
**Medical History**				
**Abnormal glycemia**	2259 (36.0)	1163 (67.0)	1096 (24.2)	< 0.01
**impaired glucose tolerance, n (%)**	191 (3.0)	54 (3.1)	137 (3.0)	0.85
**impaired fasting glucose, n (%)**	59 (0.9)	22 (1.3)	37 (0.8)	0.01
**type 1 diabetes mellitus, n (%)**	14 (0.2)	10 (0.6)	4 (0.1)	< 0.01
**type 2 diabetes mellitus, n (%)**	2004 (32.0)	1082 (62.3)	922 (20.3)	< 0.01
**Hypertension, n (%)**	4527 (72.2)	1718 (99.0)	2809 (61.9)	< 0.01
**Lipid metabolism disorder, n (%)**	2334 (37.2)	831 (47.9)	1503 (33.1)	< 0.01
**Transient ischemic attack, n (%)**	131 (2.1)	41 (2.4)	90 (2.0)	0.35
**Coronary heart disease, n (%)**	813 (13.0)	281 (16.2)	532 (11.7)	< 0.01
**Atrial fibrillation, n (%)**	364 (5.8)	81 (4.7)	283 (6.2)	0.02
**Infection within 2 weeks before admission, n (%)**	171 (2.7)	68 (3.9)	103 (2.3)	< 0.01
**Carotid artery stenosis, n (%)**	38 (0.6)	12 (0.7)	26 (0.6)	0.59
**Cancer, n (%)**	49 (0.8)	13 (0.7)	36 (0.8)	0.86
**Sleep apnea, n (%)**	48 (0.8)	32 (1.8)	16 (0.4)	< 0.01
**Smoke, n (%)**	2757 (44.0)	762 (43.9)	1995 (44.0)	0.94
**Drink, n (%)**	2827 (45.1)	835 (48.1)	1992 (43.9)	< 0.01
**mRS**				0.64
0–2, n (%)	6058 (96.6)	1680 (96.8)	4378 (96.5)	
3–5, n (%)	213 (3.4)	56 (3.2)	157 (3.5)	
**Waist, Mean ± SD, cm**	87.2 ± 11.6	96.5 ± 11.2	83.6 ± 9.6	< 0.01
**BMI, Mean ± SD, kg/m2**	24.7 ± 3.4	26.4 ± 3.5	24.1 ± 3.1	< 0.01
**HbA1c, Mean ± SD, %**	6.5 ± 1.7	7.9 ± 2.1	6.0 ± 1.3	< 0.01
**eGDR, median(IQR), mg/kg/min**	7.0 (5.8–8.6)	5.0 (4.2–5.6)	7.6 (6.8–9.6)	< 0.01
**Total cholesterol, Mean ± SD, mmol/L**	4.4 ± 1.2	4.5 ± 1.3	4.3 ± 1.2	< 0.01
Nmiss (%)	183 (2.9)	49 (2.8)	134 (3.0)	
**Low density lipoprotein, Mean ± SD, mmol/L**	2.6 ± 1.0	2.6 ± 1.1	2.6 ± 1.0	0.08
Nmiss (%)	204 (3.3)	54 (3.1)	150 (3.3)	
**High density lipoprotein, Mean ± SD, mmol/L**	1.1 ± 0.3	1.1 ± 0.3	1.1 ± 0.3	< 0.01
Nmiss (%)	203 (3.2)	54 (3.1)	149 (3.3)	
**Triglycerides, Mean ± SD, mmol/L**	1.7 ± 1.2	2.0 ± 1.6	1.6 ± 1.0	< 0.01
Nmiss (%)	196 (3.1)	53 (3.1)	143 (3.2)	
**Creatinine, Mean ± SD, μmoI/L**	72.9 ± 27.2	72.4 ± 28.5	73.1 ± 26.6	0.34
Nmiss (%)	1862 (29.7)	491 (28.3)	1371 (30.2)	
**Admission NIHSS score, Mean ± SD**	4.2 ± 3.8	4.0 ± 3.4	4.2 ± 3.9	0.78
**Admission SBP, median(IQR), mmHg**	151.4 ± 22.0	156.8 ± 21.8	149.4 ± 21.8	< 0.01
**Admission DBP, median(IQR), mmHg**	87.8 ± 13.3	90.5 ± 13.8	86.7 ± 13.0	< 0.01
**Endovascular therapy, n (%)**	35 (0.6)	9 (0.5)	26 (0.6)	0.79
**Thrombolysis, n (%)**	754 (12.0)	151 (8.7)	603 (13.3)	< 0.01
**Follow-up in 3 months**				
**antihypertensive therapy, n (%)**	3340 (53.3)	1255 (72.3)	2085 (46.0)	< 0.01
**hypoglycemic therapy, n (%)**	1660 (26.5)	929 (53.5)	731 (16.1)	< 0.01
only insulin, n (%)	317 (5.1)	179 (10.3)	138 (3.0)	< 0.01
only oral antidiabetic drugs, n (%)	1073 (17.1)	565 (32.5)	508 (11.2)	< 0.01
insulin + oral antidiabetic drugs, n (%)	270 (4.3)	185 (10.7)	85 (1.9)	< 0.01
**lipid-lowering therapy, n (%)**	5203 (83.0)	1463 (84.3)	3740 (82.5)	0.09
**antiplatelet therapy, n (%)**	5564 (88.7)	1571 (90.5)	3993 (88.0)	< 0.01
**anticoagulant therapy, n (%)**	187 (3.0)	32 (1.8)	155 (3.4)	< 0.01
**Follow-up in one year**				
**antihypertensive therapy, n (%)**	3358 (53.5)	1247 (71.8)	2111 (46.5)	< 0.01
**hypoglycemic therapy, n (%)**	1651 (26.3)	915 (52.7)	736 (16.2)	< 0.01
only insulin, n (%)	319 (5.1)	168 (9.7)	151 (3.3)	< 0.01
only oral antidiabetic drugs, n (%)	1064 (17.0)	563 (32.4)	501 (11.0)	< 0.01
insulin + oral antidiabetic drugs, n (%)	267 (4.3)	184 (10.6)	83 (1.8)	< 0.01
**lipid-lowering therapy, n (%)**	4619 (73.7)	1299 (74.8)	3320 (73.2)	0.19
**antiplatelet therapy, n (%)**	5178 (82.6)	1446 (83.3)	3732 (82.3)	0.35
**anticoagulant therapy, n (%)**	166 (2.6)	30 (1.7)	136 (3.0)	< 0.01

SD, standard deviation; IQR, interquartile range; mRS, modified Rankin Scale; NIHSS, National Institutes of Health Stroke Scale; SBP, Systolic Blood Pressure; DBP, Diastolic Blood Pressure; BMI, Body Mass Index; HbA1c, glycated hemoglobin A; eGDR, estimated glucose disposal rate

### Relationship between eGDR and study outcome at 3 months

Table 2 summarized 3-month and one-year stroke outcomes including stroke recurrence, combined vascular event and functional outcome.

**Table 2:** Univariate and multivariate analyses of outcomes between lower and higher eGDR group in AIS patients

	N(%)	Model 1*		Model 2†		Model 3‡	
		OR(95%CI)	*P-*value	OR(95%CI)	*P-*value	OR(95%CI)	*P-*value
**follow-up in 3 months**							
**stroke recurrence**							
lower eGDR group	117 (6.74)	Ref.		Ref.		Ref.	
higher eGDR group	223 (4.92)	0.72(0.58,0.91)	< 0.01	0.71(0.57,0.89)	< 0.01	0.81(0.61,1.06)	0.12
**favorable functional outcome (mRs 0–2)**							
lower eGDR group	1524 (87.79)	Ref.		Ref.		Ref.	
higher eGDR group	4031 (88.89)	1.11(0.94,1.32)	0.22	1.17(0.98,1.39)	0.08	1.16(0.94,1.43)	0.16
**excellent functional outcome (mRs 0–1)**							
lower eGDR group	1275 (73.44)	Ref.		Ref.		Ref.	
higher eGDR group	3539 (78.04)	1.28(1.13,1.46)	< 0.01	1.33(1.17,1.51)	< 0.01	1.24(1.06,1.45)	< 0.01
**follow-up in one year**							
**stroke recurrence**							
lower eGDR group	165 (9.5)	Ref.		Ref.		Ref.	
higher eGDR group	354 (7.81)	0.81(0.68,0.98)	0.03	0.80(0.67,0.96)	0.02	0.91(0.73,1.14)	0.41
**combined vascular event**							
lower eGDR group	167 (9.62)	Ref.		Ref.		Ref.	
higher eGDR group	366 (8.07)	0.83(0.69,1.00)	0.05	0.82(0.68,0.98)	0.03	0.93(0.75,1.17)	0.54
**favorable functional outcome (mRs 0–2)**							
lower eGDR group	1543 (88.88)	Ref.		Ref.		Ref.	
higher eGDR group	4109 (90.61)	1.21(1.01,1.44)	0.04	1.30(1.08,1.56)	< 0.01	1.55(1.24,1.93)	< 0.01
**excellent functional outcome (mRs 0–1)**							
lower eGDR group	1344 (77.42)	Ref.		Ref.		Ref.	
higher eGDR group	3667 (80.86)	1.23(1.08,1.41)	< 0.01	1.29(1.13,1.48)	< 0.01	1.28(1.09,1.51)	< 0.01

OR, odds ratio; eGDR, estimated glucose disposal rate; *Model 1 was unadjusted; †Model 2 was adjusted for age and sex; ‡Model 3 was adjusted for age, sex, type 1 diabetes (T1D), type 2 diabetes (T2D), lipid metabolism disorder, coronary heart disease, atrial fibrillation, infection within 2 weeks before admission, sleep apnea, drinking, body mass index (BMI), high density lipoprotein, triglycerides, thrombolysis, hypoglycemic therapy, antiplatelet therapy and anticoagulant therapy.

In univariate analysis, the rate of ischemic stroke recurrence at 3 months of lower eGDR group was significantly lower than that of higher eGDR group (4.92% vs. 6.74%; P < 0.01), and the rate of excellent functional outcome of lower eGDR group was significantly higher (78.04% vs. 73.44%; P < 0.01).

For multivariate analysis, in model 2 adjusted for age and sex, the rate of ischemic stroke recurrence at 3 months of lower eGDR group was significantly lower than that of higher eGDR group (adjusted OR 0.71[95%CI: 0.57–0.89; p < 0.01]), and the rate of excellent functional outcome was significantly higher (adjusted OR 1.33[95%CI: 1.17–1.51; p < 0.01).

**Fig. 2:**
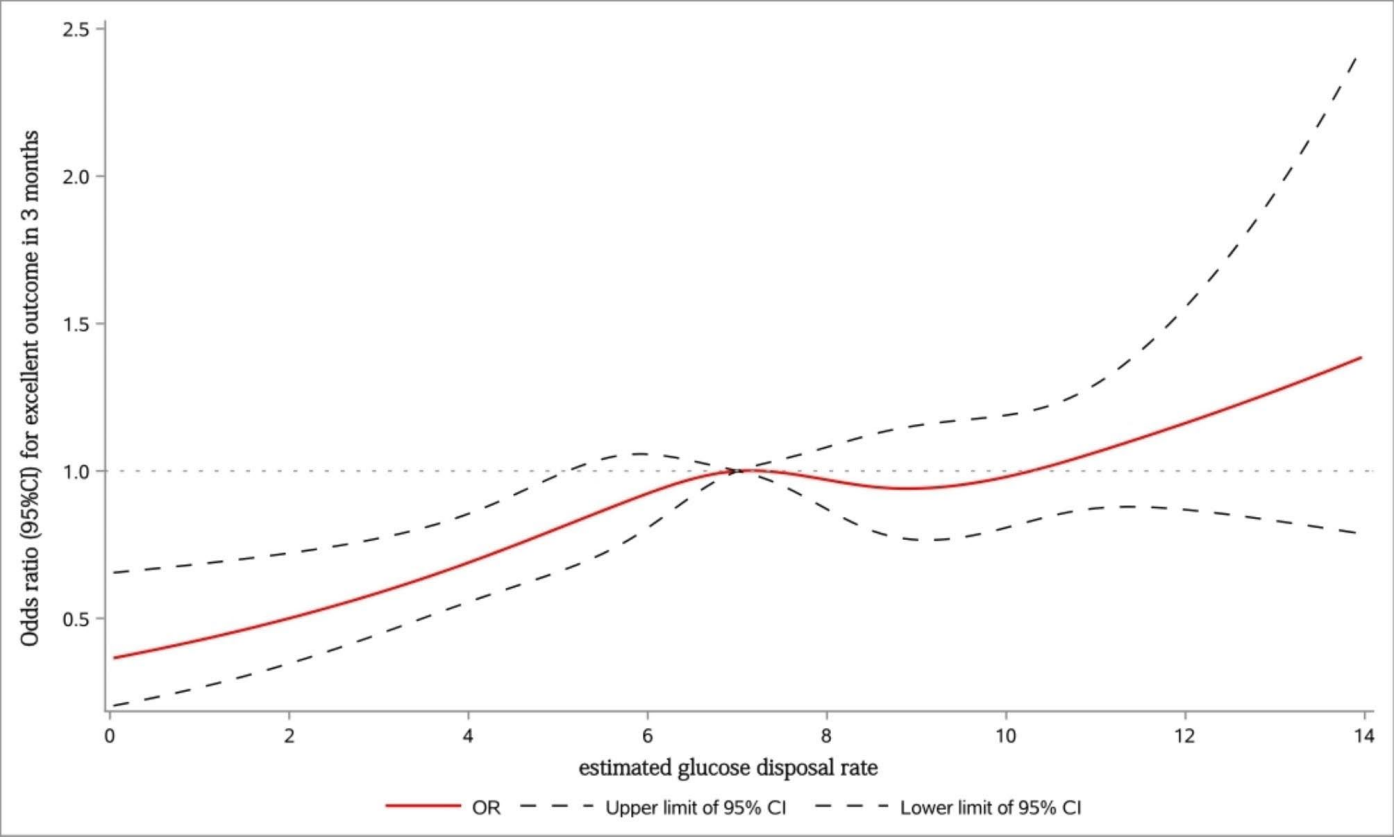
Restricted cubic spline displays the linear relation between eGDR and excellent outcome at 3 months. This figure showed the adjusted odds ratio (solid red line) and 95% confidence interval (black dashed lines) for the association between estimated glucose disposal rate and excellent outcome at 3 months. The reference level was set at 7.0 mg/kg/min. The logistic model was adjusted for age, sex and other variables (Model 3)

In model 3, after adjustment for demographic parameters and imbalanced baseline variables, OR for excellent functional outcome remained statistically significant (model 3, adjusted OR 1.36[95%CI: 1.06–1.45; p < 0.01]). There was no significant difference in the rates of recurrence (model 3, adjusted OR 0.81[95%CI: 0.61–1.06; p = 0.12]) and favorable functional outcome (model 3, adjusted OR 1.16[95%CI: 0.94–1.43; p = 0.16]) during the 3-month period. Figure 2 showed the relationship between eGDR (as a continuous variable) and excellent outcome at 3 months. When eGDR < 7.0 mg/kg/min, the relationship was approximately linear.

### Relationship between eGDR and study outcome at one year

Patients in higher eGDR group had a higher incidence of favorable functional outcomes (90.61% versus 88.88%, p = 0.04) and excellent functional outcome(80.86% versus 77.42%, p < 0.01) at one-year follow-up. These differences remained statistically significant (favorable functional outcomes, adjusted OR 1.55[95%CI: 1.24–1.93; p < 0.01]; excellent functional outcomes, adjusted OR 1.28[95%CI: 1.09–1.51; p < 0.01]) after adjusting for age, sex and other confounders.

During the one-year follow-up period, 167(9.62%) and 366(8.07%) combined vascular events occurred in lower eGDR and higher eGDR group respectively. After adjusting for confounders, no significant difference in combined vascular events (model 3, adjusted OR 0.93[95%CI: 0.75–1.17; p = 0.54]) and stroke recurrence (model 3, adjusted OR 0.91[95%CI: 0.73–1.14; p = 0.41]) was found between these two groups.

### Subgroup analysis

**Fig. 3:**
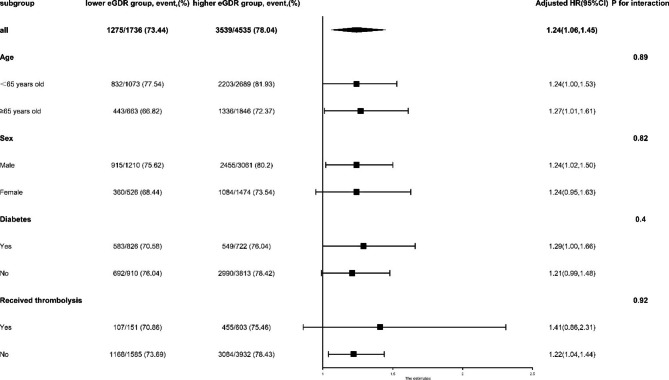
Forest plot of excellent functional outcome (mRs 0–1) in AIS patients according to different subgroups. Adjusted model included age, sex, type 1 diabetes (T1D), type 2 diabetes (T2D), lipid metabolism disorder, coronary heart disease, atrial fibrillation, infection within 2 weeks before admission, sleep apnea, drinking, body mass index (BMI), high density lipoprotein, triglycerides, thrombolysis, hypoglycemic therapy, antiplatelet therapy and anticoagulant therapy

To further validate our result, we analyzed the relationship between eGDR and excellent functional outcome at 3 months in four subgroups stratified by age, sex, diabetes and thrombolysis therapy. The results and forest plots of subgroup analyses were shown in Fig. 3. In the subgroups of female (model 3, adjusted OR 1.24[95%CI: 0.95–1.63; p = 0.11]), without diabetes (model 3, adjusted OR 1.21[95%CI: 0.99–1.48; p = 0.06]) and thrombolysis therapy (model 3, adjusted OR 1.21[95%CI: 0.99–1.48; p = 0.06]), the comparisons of outcome revealed no significant difference between two groups. However, no interaction was observed among all four subgroups. The results of other subgroups were consistent with the overall study results.

## Discussion

This nationwide large-scale study showed significant association between insulin resistance assessed by eGDR and stroke functional outcomes in first-time AIS patients. Higher eGDR was a predictor of good and excellent functional outcome independent of traditional vascular risk factors including diabetes, hyperlipidemia and atrial fibrillation.

Previously, the Fukuoka Stroke Registry reported that insulin resistance assessed by HOMA-IR was independently associated with functional outcome, but did not increase the risk of stroke recurrence at 3 months in patients with AIS [9]. In contrast, the ACROSS-China registry (Abnormal Glucose Regulation in Patients with Acute Stroke Across China) showed that HOMA-IR was an independent predictor of poor functional outcome, stroke recurrence and mortality within one year in nondiabetic patients with AIS [21]. Our results were consistent with the association between insulin resistance and functional outcome, but not with the association between insulin resistance and stroke recurrence or mortality. These findings were in line with the hypothesis that insulin resistance was detrimental to neurological recovery, and caused worse functional outcomes in AIS patients.

At present, there are several studies on the treatment of insulin resistance in stroke patients. The IRIS trial reported that treatment of insulin resistance appeared to reduce the risk of recurrent stroke. However, the reduction was not statistically different [10]. One meta-analysis including IRIS, J-SPIRIT (Juntendo Stroke Prevention study in Insulin Resistance and Impaired glucose Tolerance) [22] and PROactive (PROspective pioglitAzone Clinical Trial In macroVascular Events) [23] showed that pioglitazone could reduce the risk of recurrent stroke (hazard ratio 0.68; 95% CI, 0.50–0.92; P = 0.01) in ischemic stroke patients. The functional outcome was another important measuring marker of stroke prognosis. There is no strong evidence yet indicating the beneficial effect of treatment of insulin resistance for functional outcome after AIS. Future studies are required to investigate this hypothesis.

There are several possible explanations for poorer functional outcome in AIS patients with elevated insulin resistance. First, one study showed that patients with elevated insulin resistance had reduced hemostatic markers levels [24]. Insulin resistance may cause a procoagulant tendency in AIS patients and lead to more severe strokes. Second, Insulin resistance was associated with increased production of pro-inflammatory cytokines [25]. In patients with insulin resistance, acute inflammation which was triggered by cerebral ischemia may enhance local inflammatory and finally aggravate ischemic injury [26]. Third, insulin resistance was often accompanied by oxidative stress. This oxidative stress contributed to impairment of neurons and synaptic dysfunction [27]. Thus, it may damage the compensatory mechanisms at the recovery of stroke [28]. These possible mechanisms demonstrated that evaluation and alleviation of insulin resistance for AIS patients may be beneficial, but more specific mechanisms will be needed.

The gold standard for analyzing insulin resistance is currently hyperinsulinemic-euglycemic clamp, but it is not suitable for clinical practice and large cohort studies because of the invasiveness and cost. Most previous studies defined insulin resistance by HOMA-IR index. However, HOMA-IR index was calculated based on fasting glucose and fasting insulin. In standard clinical management of stroke patients, fasting insulin levels are not routinely tested. Besides, the measurement of HOMA-IR can be influenced by other factors such as using of insulin, insulin sensitizers, and insulin secretagogues [29, 30]. eGDR [17] is based on patient’s body size, HbA1c, and presence of hypertension, all of which are included in the routine examination upon admission of AIS patients. Therefore, it is more suitable for application in the secondary prevention of AIS patients. Many other potential insulin resistance markers like homeostasis assessment of β-cell function (HOMA-β) [31], quantitative insulin sensitivity check index (QUICKI) [32], and TyG index [33] were discovered successively, but more large-scale clinical studies are required for the extensive application of these markers.

Our study has several limitations. First, the population of our study was from hospitals in China. The distribution of etiologic subtypes of ischemic stroke in Chinese population is different from that in western population, and ischemic strokes in Chinese patients were more likely to be caused by large artery atherosclerosis. This limits the generalizability of our findings. Second, due to the large number of missing values in serum creatinine, we could not accurately evaluate the effect of renal function on stroke prognosis. Previous study demonstrated that diabetic kidney disease might mediate the effect of insulin resistance on the increased cardiovascular disease risk [34]. However, based on available data, we did not find the difference of prior renal insufficiency incidence (15 (0.9%) vs. 34 (0.7%), p = 0.65) and creatinine (73.1 ± 26.6 vs. 72.4 ± 28.5, p = 0.34) between higher and lower eGDR groups. Third, the duration of follow-up in our cohorts was relatively short. Several studies showed that assessment of eGDR effect on risk of vascular events among patients with diabetes may be more pronounced with a longer follow-up period [8, 35]. Therefore, longer follow-up is needed.

## Conclusion

eGDR is a predictor of functional outcome in patients with AIS, independent of traditional cardiovascular predictors.

### Electronic supplementary material

Below is the link to the electronic supplementary material.

Supplementary Material 1

## Data Availability

The data that support the findings of this study are available from the corresponding author but restrictions apply to the availability of these data, which were used under license for the current study, and so are not publicly available. Data are however available from the authors upon reasonable request and with permission of the corresponding author.

## Non-standard Abbreviations and Acronyms

ACROSS-China registry: Abnormal Glucose Regulation in Patients with Acute Stroke Across China
AIS: Acute ischemic stroke
BMI: Body Mass Index
CI: Confidence intervals
CNSR-III: China National Stroke Registry III
eGDR: Estimated glucose disposal rate
GFR: Glomerular filtration rate
HbA1c: Glycated hemoglobin A
HDL: High-density lipoprotein
HOMA-β: Homeostasis assessment of β-cell function
HOMA-IR: Homeostasis model assessment-insulin resistance
IRIS: Insulin Resistance Intervention after Stroke
J-SPIRIT: Juntendo Stroke Prevention study in Insulin Resistance and Impaired glucose Tolerance
mRS: Modified Rankin Scale
NIHSS: National Institutes of Health Stroke Scale
OR: Odds ratio
PROactive: PROspective pioglitAzone Clinical Trial In macroVascular Events
QUICKI: Quantitative insulin sensitivity check index
SD: Standard deviation
T1D: Type 1 diabetes
T2D: Type 2 diabetes
TIA: Transient ischemic attack
TyG index: Triglyceride glucose index
WC: Waist circumference
