# Supplementary material for: Insulin resistance estimated by estimated glucose disposal rate predicts outcomes in acute ischemic stroke patients
Source: Cardiovasc Diabetol. 2023 Aug 26;22:225. doi: 10.1186/s12933-023-01925-1 (PMC10464388; doi:10.1186/s12933-023-01925-1)
Supplement: Supplementary file 1 — Supplementary Material 1 [file 12933_2023_1925_MOESM1_ESM.docx]

| STROBE Statement—checklist of items that should be included in reports of observational studies | | | |
| --- | --- | --- | --- |
|  | **Item  No.** | **Recommendation** | **Page No.** |
|  |  |  |  |
| **Title and abstract** | 1 | (*a*) Indicate the study’s design with a commonly used term in the title or the abstract | **Page 2-3** |
|  |  | (*b*) Provide in the abstract an informative and balanced summary of what was done and what was found | **Page 2-3** |
| **Introduction** | | | |
| Background/rationale | 2 | Explain the scientific background and rationale for the investigation being reported | **Page 5-6** |
| Objectives | 3 | State specific objectives, including any prespecified hypotheses | **Page 6** |
| **Methods** | | | |
| Study design | 4 | Present key elements of study design early in the paper | **Page 7** |
| Setting | 5 | Describe the setting, locations, and relevant dates, including periods of recruitment, exposure, follow-up, and data collection | **Page 7-8** |
| Participants | 6 | Give the eligibility criteria, and the sources and methods of case ascertainment and control selection. Give the rationale for the choice of cases and controls | **Page 7** |
| Variables | 7 | Clearly define all outcomes, exposures, predictors, potential confounders, and effect modifiers. Give diagnostic criteria, if applicable | **Page 7-8** |
| Data sources/ measurement | 8* | For each variable of interest, give sources of data and details of methods of assessment (measurement). Describe comparability of assessment methods if there is more than one group | **Page 7-8** |
| Bias | 9 | Describe any efforts to address potential sources of bias | **Page 8-9** |
| Study size | 10 | Explain how the study size was arrived at | **Page 9-10** |
| Quantitative variables | 11 | Explain how quantitative variables were handled in the analyses. If applicable, describe which groupings were chosen and why | **Page 8-9** |
| Statistical methods | 12 | (*a*) Describe all statistical methods, including those used to control for confounding | **Page 8-9** |
|  |  | (*b*) Describe any methods used to examine subgroups and interactions | **Page 8-9** |
|  |  | (*c*) Explain how missing data were addressed | **Page 9** |
|  |  | *(d)*If applicable, explain how matching of cases and controls was addressed | not applicable |
|  |  | (*e*) Describe any sensitivity analyses | **Page 9** |
| **Results** |  |  |  |
| Participants | 13* | (a) Report numbers of individuals at each stage of study—eg numbers potentially eligible, examined for eligibility, confirmed eligible, included in the study, completing follow-up, and analysed | **Page 9-10** |
|  |  | (b) Give reasons for non-participation at each stage | **Page 9-10** |
|  |  | (c) Consider use of a flow diagram | **Figure 1** |
| Descriptive data | 14* | (a) Give characteristics of study participants (e.g. demographic, clinical, social) and information on exposures and potential confounders | **Page 10**  **Table 1** |
|  |  | (b) Indicate number of participants with missing data for each variable of interest | **Table 1** |
| Outcome data | 15* | Report numbers in each exposure category, or summary measures of exposure | **Page 10-12** |
| Main results | 16 | (*a*) Give unadjusted estimates and, if applicable, confounder-adjusted estimates and their precision (e.g., 95% confidence interval). Make clear which confounders were adjusted for and why they were included | **Page 10-12** |
|  |  | (*b*) Report category boundaries when continuous variables were categorized | **Table 1** |
|  |  | (*c*) If relevant, consider translating estimates of relative risk into absolute risk for a meaningful time period | **not applicable** |
| Other analyses | 17 | Report other analyses done—eg analyses of subgroups and interactions, and sensitivity analyses | **Page 12-13**  **Figure 3** |
| **Discussion** |  |  |  |
| Key results | 18 | Summarise key results with reference to study objectives | **Page 13** |
| Limitations | 19 | Discuss limitations of the study, taking into account sources of potential bias or imprecision. Discuss both direction and magnitude of any potential bias | **Page 15-16** |
| Interpretation | 20 | Give a cautious overall interpretation of results considering objectives, limitations, multiplicity of analyses, results from similar studies, and other relevant evidence | **Page 13-15** |
| Generalisability | 21 | Discuss the generalisability (external validity) of the study results | **Page 16** |
| **Other information** | |  |  |
| Funding | 22 | Give the source of funding and the role of the funders for the present study and, if applicable, for the original study on which the present article is based | **Page 17** |
